# Integrating Human Papillomavirus and HIV Services Into a One-Stop Breast Cancer Early Detection Clinic in Zambia

**DOI:** 10.1200/GO-25-00547

**Published:** 2026-04-03

**Authors:** Mutumba Songiso, Anna Cabanes, Mpimpa Mutale, Mulindi Mwanahamuntu, Ronda Henry-Tillman, Groesbeck P. Parham

**Affiliations:** ^1^ Levy Mwanawasa University Teaching Hospital, Lusaka, Zambia; ^2^ Levy Mwanawasa Medical University, Lusaka, Zambia; ^3^ Global Focus on Cancer, New York, NY; ^4^ University of Lusaka, Department of Physiological Sciences, Lusaka, Zambia; ^5^ University Teaching Hospital, Women and Newborn Hospital, Lusaka, Zambia; ^6^ University of Arkansas for Medical Science, Little Rock, AK

## Abstract

**PURPOSE:**

Women in low- and middle-income countries face overlapping burdens of breast cancer, cervical cancer, and HIV. Fragmented service delivery contributes to delayed prevention, missed diagnoses, and inefficient use of scarce health resources. We evaluated the feasibility and acceptability of an integrated service delivery model in which HIV and human papillomavirus (HPV) testing were incorporated into an established One-Stop Breast Care Clinic at the primary care level in Zambia.

**METHODS:**

Between October 2021 and March 2022, women attending the breast clinic were recruited using convenience sampling. Before breast assessment, participants were offered HIV testing and instructed in HPV self-collection. Samples were processed onsite, in the facility's laboratory, and referrals were guided by test results. Patient satisfaction was assessed through structured questionnaires. Feasibility, acceptability, and implementation challenges were explored through semi-structured interviews with hospital administrators.

**RESULTS:**

Among 180 participants (the majority <55 years), 23.3% tested positive for HPV and 18.9% for HIV; all were referred for immediate follow-up care. Breast evaluations identified benign disease in 86.7% and malignancy in 13.3%. Overall satisfaction with receiving integrated HIV and HPV services during breast evaluation was high (97.2%). Satisfaction with clinic flow was reported as satisfactory by 63.9% and highly satisfactory by 33.9% of participants. Hospital administrators highlighted improved efficiency and patient-centeredness of the integrated model. The principal barrier to sustainability was the cost of HPV testing.

**CONCLUSION:**

Integration of cervical cancer screening, breast cancer early detection, and HIV services into a single women-centered clinic at the primary care level is feasible, acceptable, and resource-efficient. This model aligns with global elimination and early detection targets and offers a scalable approach to strengthen women's health services in resource-limited settings.

## INTRODUCTION

Each year, approximately 3 million women worldwide are diagnosed with breast or cervical cancer, and an estimated 1 million die from these diseases.^1^ Together, these two malignancies account for nearly half of all cancers diagnosed among women globally. Breast cancer was the most frequently diagnosed cancer in women in 2022 (2.3 million new cases; 666,103 deaths), whereas cervical cancer ranked fourth with 660,000 new cases and 350,000 deaths.^1^

**CONTEXT**

**Key Objective**
This study illustrates that service integration is particularly beneficial in resource-limited environments by enabling multiple screenings to be conducted at a single site. The clinic successfully combined health education with onsite HIV and human papillomavirus (HPV) testing and breast care assessments, achieving a 97% participant satisfaction rate.
**Knowledge Generated**
The implementation of HPV self-sampling and rapid HIV testing addressed privacy and discomfort concerns, reduced staffing requirements, and improved both operational efficiency and patient experiences. This integrated model facilitated the detection of previously unrecognized cases of HPV and HIV, resulting in timely referrals and helping to avert disease progression. Both patients and hospital administrators strongly supported the initiative, although challenges, such as the high costs associated with HPV testing technologies, were noted.
**Relevance**
These findings underscore the benefits of patient-centered, integrated cancer screening approaches, while also highlighting substantial structural, financial, and workforce barriers that must be addressed to support the expansion and sustainability of women's cancer prevention and early detection programs in Zambia and similar contexts.

The burden is particularly high in sub-Saharan Africa (SSA), with 133,520 breast and 117,944 cervical cancer cases reported in 2022.^1,2^ Cervical cancer remains the leading cause of cancer-related mortality among women in the region (76,140 deaths), followed closely by breast cancer (68,036 deaths).^1^

Zambia faces some of the highest cervical cancer rates in the world, with an incidence of 71.5/100,000 and a mortality rate of 49.4/100,000.^3^ Together, cervical and breast cancers account for approximately 53.6% of all cancer cases among Zambian women.^1^

As both the number and life expectancy of women living with HIV (WLHIV) increase, and as the incidence of cervical and breast cancer continues to rise, the intersection between HIV and women's cancers has become increasingly significant.^4,5^ WLHIV are five to six times more likely to develop invasive cervical cancer than HIV-negative women, particularly in regions where screening programs are limited.^4^ The proportion of cancers attributable to HIV varies globally; in Africa, cervical cancer alone accounts for 40.8% (23,400 of 57,300) of all HIV-associated cancers.^5^

Emerging evidence also suggests a growing burden of breast cancer among WLHIV.^6^ In SSA, one in four women under age 50 years diagnosed with breast cancer is coinfected with HIV. The incidence of breast cancer in WLHIV continues to increase. In Zambia, the HIV prevalence among adults age 15-59 years is 12.3%, rising to 14.9% among females.^7^ This corresponds to approximately 980,000 adults living with HIV in this age group.^7^ Although national HIV incidence is declining—reflecting progress toward epidemic control—incidence among women remains unacceptably high.^7,8^ The growing convergence of HIV, cervical, and breast cancers underscores the urgent need to address these diseases together through integrated prevention, screening, treatment, and care strategies.

In March 2018, in alignment with the Zambian Ministry of Health's strategy to decentralize cancer services, the Matero Breast Care Specialty Clinic (MBCSC)—the first dedicated breast care clinic integrated within a district-level hospital in Zambia—was established at Matero Hospital.^9^ Located in the capital city of Lusaka, the MBCSC was designed to promote early detection of breast cancer by reducing delays between presentation, diagnosis, and treatment initiation in symptomatic women. The clinic provides same-day services, including breast self-awareness education, clinical breast examination (CBE), and ultrasound-guided breast biopsy. Since its inception, the clinic has evaluated over 50,000 women, with a significant increase in the proportion presenting with early-stage disease and improved 3-year survival.^10^

The Cervical Cancer Prevention Program in Zambia, established in 2005, currently operates over 100 screening sites nationwide, including Matero Hospital.^11^ WLHIV who test human papillomavirus (HPV)–positive undergo immediate treatment for cervical precancer (screen and treat) without waiting for confirmatory histology, provided they meet the eligibility criteria.^12-16^ Matero Hospital also offers same-day HIV testing and treatment initiation.

Building on these platforms, we integrated HPV-based cervical cancer screening and HIV testing into an existing MBCSC to enhance service accessibility, minimize patient dropout, and improve overall patient satisfaction.

## METHODS

### Study Setting and Population

The study was conducted at Matero Hospital in Lusaka, a district-level hospital serving an estimated population of 480,000 people. Women age 25 years and older presenting with breast symptoms at the MBCSC were eligible for enrollment.

### Ethical Consideration

The study protocol was reviewed and approved by the University of Zambia Biomedical Research Ethics Committee (UNZABREC; Ref. No. 2036-2021) and the National Health Research Ethics Committee (NHREC; Ref No: NHRA0006/29/09/2021). Written informed consent was obtained from all participants.

### Participant Recruitment

**FIG 1:**
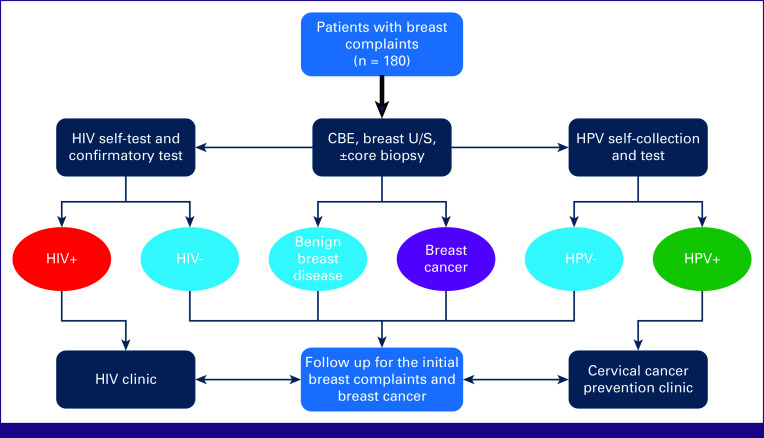
Recruitment and flow of patients. CBE, clinical breast examination; HPV, human; U/S, ultrasound.

Between October 2021 and March 2022, 180 women were enrolled using convenience sampling at the time of presentation to the MBCSC. Only two patients chose not to participate in the study for personal reasons, whereas all other eligible patients were enrolled consecutively (Fig 1).

### Clinical Procedures

The standard clinical protocol was as follows: Before breast evaluation, women age ≥25 years presenting to the Matero Breast Care Specialty (MBCSC), and who had not undergone cervical cancer screening within the preceding 12 months, were offered HPV testing using self-collected vaginal samples. Specimens were immediately transported to the onsite laboratory and tested for oncogenic HPV using the GeneXpert platform. Women who had never tested for HIV, or whose most recent negative test was more than 6 months before, were offered HIV testing using OraQuick self-test kits. Those with reactive results underwent immediate blood-based rapid confirmatory testing onsite. Following HPV and HIV testing participants then underwent a CBE by a trained health care professional. Suspicious findings (eg, breast masses, bloody nipple discharge, breast asymmetry) were further evaluated by a breast surgeon. When indicated, ultrasound-guided core needle biopsy was performed on the same day.

The patients received their HIV results on the same day the test was performed in the clinic. For HPV testing, the patient collected their own sample at the breast clinic, but the analysis took place in the laboratory. Since the lab had only one GeneXpert machine—which was primarily used for TB specimens—the HPV samples were tested later in the afternoon, only after all the TB samples had been processed. Patients with positive HPV results received their results at the following clinic visit and were subsequently referred to the cervical cancer clinic on the same day, where they received treatment immediately. Treatment was provided using thermal ablation or large loop excision of the transformation zone (LLETZ), in accordance with national cervical cancer prevention guidelines. Women with clinical findings suspicious for invasive cervical cancer underwent cervical biopsy and were referred to the gynecologic oncology clinic at the University Teaching Hospital (UTH) in Lusaka. Biopsy and LLETZ specimens were transported daily by courier to the UTH Pathology Laboratory. Patients were followed according to established national protocols. Women with confirmed HIV infection were referred to the Matero HIV Clinic for same-day antiretroviral therapy initiation and care navigation.

### Data Analysis

Quantitative data were analyzed descriptively, with frequencies and percentages used to summarize key variables and presented in tabular format. Qualitative data, from open-ended questionnaire responses, were analyzed thematically to identify common patterns related to patient experiences, satisfaction, and recommendations for service improvement (Appendix Table A1).

### Process Evaluation of the One-Stop Women's Clinic

Between months 6 and 12 of implementation, individual interviews were conducted with clinic users, service providers, and hospital administrators to assess the acceptability, feasibility, and implementation barriers and facilitators of the One-Stop Women's Clinic model.

Separate semi-structured interview guides were developed for clinic users and for service providers and administrators, informed by five domains of the Consolidated Framework for Implementation Research: intervention characteristics, outer setting, inner setting, characteristics of individuals involved, and implementation process.^17^

### Interviews With Participating Women

Women who had accepted all three services (breast evaluation, HPV testing, and HIV testing) were eligible for interviews and were contacted within 3 months of their clinic visit. After providing informed consent, participants completed interviewer-administered questionnaires capturing demographic characteristics, referral pathways, satisfaction with clinic services, and suggestions for improvement. Interviews were conducted by trained clinic staff in the participant's preferred language (local language or English).

The questionnaire gathered information about how patients were referred to the clinic, their satisfaction with how services were provided, the respect shown by providers, and the overall quality of care. It also asked about patients' comfort with having HIV and HPV testing done during breast evaluations and whether they would recommend the clinic to others. Additionally, the survey included open-ended questions for patients to share their experiences and offer suggestions for improvement.

## RESULTS

### Participant Characteristics

A total of 180 women were included in the study (Table 1). The largest age group was 25-34 years (40.6%), followed by 35-44 years (31.1%), 45-54 years (20.6%), and >54 years (7.8%). Nearly half of participants had completed secondary education (47.2%), whereas 22.8% had primary education and 30.0% had tertiary education. Most women resided within Lusaka city but outside Matero township (65.6%); 23.9% lived within Matero township, and 10.6% lived outside Lusaka.

**TABLE 1:** Demographics of Participants

Variable	Values	Frequency, No. (%)
Ages of participants, years	25-34	73 (40.6)
	35-44	56 (31.1)
	45-54	37 (20.6)
	>54	14 (7.8)
Education level attained	Primary	41 (22.8)
	Secondary	85 (47.2)
	Tertiary	54 (30.0)
Residential area where participants resided	Within Matero	43 (23.9)
	Within Lusaka but >5 km outside of Matero	118 (65.6)
	Outside Lusaka	19 (10.6)

HPV testing identified 42 women (23.3%) as HPV positive, whereas 138 (76.7%) tested negative. HIV prevalence was 18.9% (34 participants), of whom 23 were already receiving antiretroviral therapy. HIV-HPV coinfection was observed in 15 of the 42 HPV-positive women (36%). Breast biopsy results demonstrated benign disease in 156 participants (86.7%) and malignancy in 24 (13.3%) as shown in Table 2.

**TABLE 2:** Test Results for HPV, HIV, and Breast Biopsy

Variable	Values	Frequency, No. (%)
HPV results	Positive	42 (23.3)
	Negative	138 (76.7)
HIV results	Positive	34 (18.9)
	Negative	146 (81.1)
Breast histopathology results	Benign breast disease	156 (86.7)
	Breast cancer	24 (13.3)

Abbreviation: HPV, human papillomavirus.

Among women who tested HPV positive, 39 (92.8%) underwent further evaluation and treatment at the Matero Cervical Cancer Prevention Clinic; treatment modalities are summarized in Table 3. Three women declined further evaluation and did not receive treatment.

**TABLE 3:** Treatment of Human Papillomavirus–Positive Participants Following Digital Cervicography

Number	Treatment Type
36	Thermal coagulation
3	LEEP/LLETZ
3	Declined treatment

Abbreviations: LEEP, loop electrosurgical excision procedure; LLETZ, large loop excision of the transformation zone.

Half of all participants (50%) learned about MBCSC through referrals from another health facility, whereas 21.1% were advised by a friend or relative, 27.2% attended for routine health services, and 1.7% learned about the clinic through radio or social media (Table 4).

**TABLE 4:** How Participants Came to Know About Matero Breast Care Specialty Clinic

How the Participant Learned About MBCSC	Frequency, No. (%)
Referred from another health care facility	90 (50.0)
Came seeking routine health services	49 (27.2)
Recommended by a friend or relative	38 (21.1)
Heard about the clinic on the radio or social media platforms	3 (1.7)
Total	180 (100.0)

Abbreviation: MBCSC, Matero Breast Care Specialty Clinic.

Table 5 provides an overview of participants' satisfaction with the services offered at the MBCSC. A substantial proportion (97.2%) reported satisfaction with the availability of HIV and HPV testing within the breast clinic. With respect to service flow, 63.9% indicated satisfaction and 33.9% were extremely satisfied; only 2.3% expressed dissatisfaction. The vast majority of respondents (99.4%) characterized provider conduct as respectful. Regarding overall care, 55.6% rated it as very good, whereas 44.4% considered it good. Additionally, 60.0% stated they were very likely to recommend the clinic to others, and 39.4% responded likely.

**TABLE 5:** Participants' Level of Satisfaction With the Services at MBCSC

Variable	Values	Frequency, No. (%)
Were participants happy being able to access HIV and HPV tests within the breast clinic setting?	Yes	175 (97.2)
	No	5 (2.8)
Level of participants' satisfaction with the flow of services offered within the breast clinic	Extremely dissatisfied	1 (.6)
	Dissatisfied	3 (1.7)
	Satisfied	115 (63.9)
	Extremely satisfied	61 (33.9)
How participants rated the level of respect demonstrated by providers to them	Respectful	179 (99.4)
	Disrespectful	1 (.6)
Participants' overall rating of the care they received during their visit	Very poor	0 (0)
	Poor	0 (0)
	Good	80 (44.4)
	Very good	100 (55.6)
The likelihood of the participant recommending the breast clinic to others^a^	Unlikely	0 (0)
	Likely	71 (39.4)
	Very likely	108 (60.0)

Abbreviations: HPV, human papillomavirus; MBCSC, Matero Breast Care Specialty Clinic.

^a^ One participant did not answer the last question on likelihood…

Finally, participants were asked whether they had any additional feedback regarding the MBCSC. The majority expressed satisfaction with the services provided and encouraged the government to continue supporting the program.

Several participants stated that they would recommend the MBCSC to others for screening and treatment. Below are selected quotes illustrating their experiences:

- *I recommend all the staff working in this department; very friendly and welcoming. I appreciate their services, and they must continue…*
- *I visited Matero Level One Hospital. They are very good and they welcome people well. They have respect and are kind to the people that they attend to. They should keep it up with the same spirit*
- *The breast clinic is good because it gives us a direction on how and when to treat cancer and avoid unnecessary deaths*
- *The clinic is doing very well, and they are taking good care of the patients and educating people about their health. Otherwise, we are happy with their services*.

Despite the overall positive feedback, some participants highlighted challenges, including long waiting times, insufficient equipment (such as mammography units), and a lengthy screening process. To address these concerns, they proposed the following recommendations:

- Extending clinic operating hours
- Expanding the screening program to rural areas
- Increasing staffing levels at the Matero Breast Clinic
- Equipping all health care facilities with mammography units to reduce the need for referrals
- Improving the clinic's infrastructure
- Enhancing public awareness campaigns on breast cancer and HPV testing
- Increasing clinic days to three per week at the Matero Breast Clinic
- These insights highlight both the strengths of the clinic and areas for improvement, emphasizing the need for continued investment in breast cancer screening and care services.

### Interviews With Health Care Providers and Hospital Administrators

#### Characteristics of the Individuals and Outer Setting

We conducted individual interviews with three hospital administrators, a head of clinical care, a health system strengthening nurse, and two nurses from the MBCSC (n = 5), the former three being administrators. All interviewees were female, age between 30 and 59 years, and held administrative or clinical positions at Matero Hospital. All had direct experience with the MBCSC.

#### Intervention Characteristics

There was consensus among administrator interviewees (3/3) that the program was well-developed and effectively integrated into Matero's existing patient workflow. One participant highlighted that the initiative leveraged the hospital's preexisting infrastructure to build the One-Stop Women's Clinic, thereby ensuring seamless service delivery. Another noted that the program effectively addressed a critical need in the community. The perceived success of the clinic was reinforced by high levels of patient satisfaction, with women not only using the services but also referring others to the program.

All five interviewees agreed that sufficient training had been provided before program implementation. Notably, the nurse reported feeling empowered by the training, as she is now able to educate patients about cancer, dispel myths (eg, misconceptions such as money placed within the bra being worn causing cancer), address stigma, and guide patients through the treatment journey.

A key strength of the clinic, as unanimously recognized by interviewees, was its ability to offer three essential services—HPV testing, HIV testing, and breast cancer screening—under one roof. Two participants reflected on the preclinic scenario, noting that the absence of such an integrated service model had led to undiagnosed breast cancer cases. Additionally, one participant among the administrators (1/3) highlighted that, in the preexisting cervical cancer screening clinic at Matero, delays in obtaining test results were common, and in some cases, results were unavailable altogether. This issue has significantly improved after the establishment of the One-Stop Women's Clinic.

During the pilot phase, the program was fully funded by a grant, ensuring that Matero Level One Hospital did not incur additional costs. However, interviewees suggested several areas for further improvement. Two nurses and one administrator emphasized the need to expand the clinic's services to include treatment options such as chemotherapy and radiation therapy. Additionally, the two nurses highlighted the necessity of integrating mammography into the clinic's diagnostic offerings. Three administrators identified a critical need for a larger space to accommodate service delivery and stressed the importance of a private area for counseling.

### Inner Setting

A key challenge identified by the three administrators was the cost of HPV DNA cartridges, which remains a limiting factor for the continued provision of HPV-based screening services. Although HIV testing and breast cancer early detection remain financially viable for the hospital, the high cost of HPV testing poses sustainability concerns.

When asked about potential improvements, there was unanimous agreement that expanding the clinic's physical space would enhance patient comfort and service efficiency.

The program has been well received within the hospital, with administrators recognizing its efficiency in optimizing hospital resources while respecting patients' time. By consolidating multiple screening and testing services in one location, the clinic has eliminated the need for women to navigate multiple hospital departments, thereby streamlining care and reducing patient burden.

## DISCUSSION

This study demonstrates the feasibility, acceptability, and value of integrating breast cancer early detection with HPV-based cervical cancer screening and HIV testing within a single primary-level clinic in Zambia. Patient satisfaction was exceptionally high, particularly regarding the convenience and comprehensiveness of integrated services. These findings align with evidence from Uganda and other settings demonstrating high acceptability of integrated HIV and cervical cancer screening.^18^

Although integration of screening and treatment for women's cancers—especially cervical and breast cancer—has been widely advocated, implementation remains challenging in many health care settings.^19,20^ Key obstacles include differing age distributions, the need for multiple clinical specialties, and management across separate hospital departments.

Notably, 23% of women presenting with breast symptoms were newly identified as HPV positive. Of the 34 patients with HIV, 23 (68%) were already receiving antiretroviral therapy and were therefore aware of their HIV status, whereas 11 (32%) were unaware of their status at the time of their breast clinic visit. These proportions are consistent with a recent study indicating that 72.3% of HIV-positive adults in Zambia knew their HIV status, whereas 27.7% remained unaware during the survey period.^21^ Onsite, same-day testing enabled prompt diagnosis, initiation of treatment, and linkage to care, highlighting the significant clinical benefits of an integrated service delivery approach.

A key factor contributing to the success of the One-Stop Clinic model was the use of HPV self-sampling and rapid oral HIV testing. By allowing women to collect samples themselves, the clinic reduced barriers associated with discomfort, privacy concerns, and the need for specialized personnel while maintaining high test accuracy. Rapid onsite processing of samples for HIV and HPV testing enabled prompt communication of results, timely diagnosis, and immediate linkage to care, potentially preventing progression of disease. This streamlined approach not only enhanced patient satisfaction but also improved clinic efficiency, demonstrating the practical value of integrating user-friendly, rapid diagnostics into comprehensive women's health services.

Participant satisfaction with the One-Stop Clinic model was high, with 97% reporting satisfaction with onsite HIV and HPV testing. Many highlighted the convenience of receiving multiple services in a single visit, avoiding the need to navigate different hospital departments. Hospital administrators also supported integration, although they noted sustainability concerns, particularly the high cost of HPV testing using the GeneXpert system.

More than 70% of participants were under age 44 years, reflecting the younger age distribution of breast and cervical cancer in Zambia and across SSA.^10^ This age overlap presents a critical opportunity to co-locate services and improve efficiency while reducing patient burden.

Despite these successes, sustainability challenges—particularly the cost of HPV testing—remain a key concern. Addressing infrastructure limitations, workforce capacity, and rural access will be essential for scale-up. Participants' calls for expanded outreach and public awareness further highlight the need to extend integrated screening beyond urban centers.

In conclusion, the One-Stop Women's Clinic model offers a scalable, patient-centered approach to addressing the intersecting burdens of HIV, cervical cancer, and breast cancer. Strategic investments to overcome resource constraints could enable broader implementation and contribute meaningfully to women's cancer control efforts in Zambia and similar settings.

## Data Availability

A data sharing statement provided by the authors is available with this article at DOI https://doi.org/10.1200/GO-25-00547.

## APPENDIX

**TABLE A1:** Elected Qualitative Quotes Illustrating Consolidated Framework for Implementation Research Domains Across the Inner Setting, Outer Setting, and Intervention Characteristics

Intervention Characteristic	Quote
General impression about the clinic	“I like the fact that they're able to get the investigations done in in in one space…three investigations done in one space. They don't need to move around. So, I like that idea that everything is being done in one space and they're able to know their HIV status and know how they are with their breast, as well as the HPV, which usually HPV is something that women don't really look for. So, it would take time for them to even, investigate for that. But the fact that it's being done in one space, it's a very good thing. I think women are happy.” (ADMIN 2)
Intervention source and evidence strength and quality	“I think the development of the program was well done because everything starting from the human resource that was needed and the commodity that was needed to carry out to this service were made available and the tools were integrated in the system so that the flow of clients was not disrupted.” (ADMIN 3)
Relative advantage: How does this type of intervention compare with the existing similar programs?	“I think it has advantages. One of the advantages that it's being offered under one roof. I think a patient doesn't have to move from one room to the other. So, I think that's what makes it viable.” (ADMIN 1)
Do you think the nurses need more training?	“The trainings that we had also helped us. We came to learn a lot of things, even things that I didn't know. And looking at that program, we have been receiving a lot of patients from different hospitals.” (HCP 2)
Inner setting	
Do you think there's enough available resources within the hospital for this program to continue when the project ends?	“So as far as HIV screening is concerned and maybe the breast cancer screening is concerned, the hospital might be able to do these on their own. But when it comes to HPV, I don't think so, because even when we implemented HPV screening before it, we've had issues in terms of stock outs of kits and turnaround time for results. So, if we needed it to be successful, I think the hospital might need more help when it comes to the HPV screening kits.” (ADMIN 3)
Structure of the hospital facilities	“Further expansion of the room or even another place could be identified for that service to continue. Big enough rooms where all services can be housed. Maybe they can be a waiting area and a separate counseling room. Facilities.” (ADMIN 1)
Adaptability: do you think this program can work in other hospitals?	“Since this is a study, I think maybe it should be incorporated in the government health system…but I think it's something that needs to be done in more places so that, our women are investigated so that they don't die from breast cancer when it is something that we can treat if found early.” (ADMIN 2)
How do people that you interact within the hospital perceive the intervention?	“Because I think most of the workers have seen the trends. Maybe ever since the opening of the breast clinic, we've seen the benefits of having that clinic within the hospital. And so, people have come to appreciate, including even some members of staff, have gone through that clinic as patients. So, I feel people have welcomed it and it can still be co-opted within the services that have been offered in the hospital.” (ADMIN 1)
Cost	“As HIV screening and the breast cancer screening is concerned, the hospital might be able to do these on their own. But when it comes to HPV, I don't think so, because even when we implemented HPV screening before it, we've had issues in terms of stockouts of kits and turnaround time for results. So, if we needed it to be successful, I think the hospital might need more help when it comes to the HPV screening kits.” (ADMIN 3)
Outer setting	
Patient needs and preferences	“Yeah, you know, adding HIV and HPV, the women are very happy, and we are also happy as well because we are doing many things in one department… And again, when they go to do these cervical screening, they don't see the HPV because it's a virus. You can see it with your naked eyes. But us we were able to do the GeneXpert test. We helped a lot of women to know if they have the HPV or not. And we were able to send them for treatment, which was a very good thing, which is a very good thing. And women are very much happy and would love to continue with this program.” (HCP 2)
Community needs	“In the sense that most people, when they knew about the breast clinic, they started coming to Matero. In fact, from all parts of the country and those who survived from breast cancer, they came back and appreciated us. And they felt that we should continue with the program to help other women out there in the community. (HCP1)

Abbreviation: HPV, human papillomavirus.
